# How to use fitness landscape models for the analysis of collective decision-making: a case of theory-transfer and its limitations

**DOI:** 10.1007/s10539-018-9669-4

**Published:** 2019-01-12

**Authors:** Peter Marks, Lasse Gerrits, Johannes Marx

**Affiliations:** 1 Department of Public Administration and Sociology, Erasmus University Rotterdam, Room T17-03, P.O. Box 1738, 3000 DR Rotterdam, The Netherlands; 2 Otto-Friedrich University, Bamberg, Germany

**Keywords:** Knowledge transfer, Fitness landscapes, Collective decision-making

## Abstract

There is considerable correspondence between theories and models used in biology and the social sciences. One type of model that is in use in both biology and the social sciences is the fitness landscape model. The properties of the fitness landscape model have been applied rather freely in the social domain. This is partly due to the versatility of the model, but it is also due to the difficulties of transferring a model to another domain. We will demonstrate that in order to transfer the biological fitness landscape model to the social science it needs to be substantially modified. We argue that the syntactic structure of the model can remain unaltered, whilst the semantic dimension requires considerable modification in order to fit the specific phenomena in the social sciences. We will first discuss the origin as well as the basic properties of the model. Subsequently, we will demonstrate the considerations and modifications pertaining to such a transfer by showing how and why we altered the model to analyse collective decision-making processes. We will demonstrate that the properties of the target domain allow for a transfer of the syntactic structure but don’t tolerate the semantic transfer.

## Introduction

There is considerable correspondence between theories and concepts used in biology and in the social sciences (e.g. Sanderson 1990). For example, Evolutionary Stable Strategies (Maynard-Smith 1982; Maynard-Smith and Price 1973) have utility in both domains. In biology, it helps focusing on the outcomes of evolution under the condition that species are primarily concerned with self-interest by Darwinian fitness. In the social sciences, it helps modeling and explaining why actors would want to engage in repeated interaction of the same kind (Axelrod 1984) and how norms could arise from that repeated interaction (Axelrod 1986; Skyrms 2014).

One type of model that is in use in both biology and in the social sciences is that of the fitness landscape. The origin of fitness landscapes can be traced back to Sewall Wright’s shifting balance theory (1931). Since then it has sparked many varieties in which the original theory has been adopted and transformed, especially by biologists such as Gavrilets (1997, 2003, 2004), Kimura (1968), and Mayr (1963) to name just a few. This diversity of interpretations and applications notwithstanding, it is especially Stuart Kauffman’s ‘The Origin of Order’ from 1993 that inspired social scientists to adopt the model to their realm (Gerrits and Marks 2014, 2015). Gerrits and Marks (2014) reviewed 163 applications of fitness landscapes in different subdomains within the social sciences. In this review, it is demonstrated that the applications vary considerably in purpose and scope, ranging from metaphors to qualitative case studies.

The upshot is that the properties and functions of fitness landscapes are attributed rather freely in the social sciences. This may be due to the model’s versatility but can also be a telltale sign of the difficulties of transferring such a model from one domain to the other. Within the social sciences, we observed that most of the applications are focused on modeling and that the number of instances in which empirical data was processed is relatively limited. Possibly due to the challenges of transferring and operationalizing the model, the number of applications using qualitative data is even smaller. These challenges notwithstanding, the whole process of transfer, operationalization and application to empirical data, especially that of a qualitative kind, can, and should, be made more relevant to the social sciences (Gerrits and Marks 2015: 472–473; Knuuttila 2011). We will argue that the differences between the two domains require substantial modifications to the original model during the transfer of the model. Following Weisberg (2007) it is argued that the evolutionary biological fitness landscapes cannot be understood as abstract direct representations [ADR] of social phenomena but could be used as an instrument for theorizing about said phenomena. We will demonstrate the dynamics of the theory transfer by showing how fitness landscapes can be used to analyze collective-decision making processes on the basis of real, and often fuzzy, empirical data.

The “Fitness landscapes in biology” section will introduce the main concept and its adoption in the social sciences. We will focus on the work by Kauffman as this has been the main source for adaptations in the social sciences. The “Transfer” section will focus on theory transfer itself. The considerations from that section will be used to demonstrate an application of the model to the analysis of collective decision-making processes itself, in the “Transfer of the original model” section.

## Fitness landscapes in biology

In 1931 Sewall Wright wrote a paper entitled ‘Evolution in Mendelian populations’, which was partially based on earlier work on population genetics (Bacaër 2010). For this paper, Wright relied on mathematics to demonstrate that new gene combinations could emerge if selection is sufficiently slight. Alleles and genotypes could change in response to evolutionary pressures such as natural selection, mutation and migration (Gavrilets 2004; McCandlish 2011). An invitation to present his ideas at a conference meant that he had to reconsider the mode of presentation, or risk running out of time with an audience who would perhaps get lost in the mathematical formulations. He therefore decided to explain the main properties of this shifting balance theory in a narrative, in terms of fields of gene combinations, and to deploy the metaphor of the ‘adaptive surface’ (Petkov 2015; Ruse 1990, 1996). To this end, he developed a number of visual representations to highlight various possibilities for the adaptive surface. These were published in the conference proceedings as ‘The roles of mutation, inbreeding, crossbreeding and selection in evolution’ (Wright 1932).

Those visual representations of his shifting balance theory later become known as the fitness landscapes or fitness landscape models. The basics of Wright’s model are relatively straightforward. It features a set of genes that occurs in combination with other genes. Assigning values to each genotype enabled Wright to represent the distribution of adaptive values under a particular set of conditions over the space of genotypes in a two-dimensional field of gene combinations. The location of those gene combinations in the adaptive surface is associated with a degree of biological fitness. In the third dimension, this fitness can be represented by peaks and lack of fitness by valleys on a surface plot. Such plots resemble (mountainous) landscapes visually. This constitutes a surface plot, or a landscape, as it became more commonly known. In such a landscape, genes will cluster around peaks because gene combinations cannot sustain low fitness owing to selection pressure.

Over the many years since it was first published, the model has been used, extended, amended, and revised as researchers discovered its possibilities and limitations (e.g. Altenberg 1995, 1997; Dobzhansky 1982; and many others). Since many decades, the plausibility of the shifting balance theory has been questioned (Coyne et al. 1997; Gavrilets 1997, 2003, 2004; Plutynski 2008; Provine 1986). For instance, Coyne et al. (1997) show that shifting balance is an efficient mechanism for adaptation only under restrictive conditions. Even Wright himself explored how its limitations influenced the kind of conclusions about selection one can draw (Wright 1968). In other words, the model is not as unambiguous as it may appear to those not involved in biology (Petkov 2014, 2015).

### The appeal of Kauffman’s fitness landscapes

The adoption into the social sciences shows a wide diversity of applications. Some authors utilize the mathematical dimension for modelling purposes, others focus on the visual representation, and yet others take more freedom in their interpretation and use it as a metaphor about peaks and adaptive walks. The most cited source for those models is not Wright’s work, but rather ‘The origin of Order’ by Stuart Kauffman (1993). The main theme of this book is a search for an explanation of the origins of life and subsequent speciation. To this end, he deploys a wide range of tools, models and concepts, with a central role for self-organization and coevolution. While some parts of the book feature confirmed theoretical knowledge, the text is also highly speculative and untestable for a considerable part (Fox 1993), and sometimes confusing and inexact (Alberch 1994; Weber 1998). The book probed biologists to look at evolution in a particular way, but it does not provide a grand theory of evolution or a coherent set of proven causal relations. Although ‘The Origins of Order’ received a mixed response in biology, owing to aforementioned reasons, it has turned into a preferred reference for social scientists using fitness landscapes for their particular research.

Kauffman attempts to model self-organization and selection in such a way that one can investigate how said self-organization enables or restricts natural selection. Fitness landscapes play a central role in this modeling attempt. Indeed, the fitness landscapes are the “conceptual glue” (Weber 1998: 135) that keeps the many arguments in the book together. These fitness landscapes are principally not very different from Wright’s adaptive landscapes but Kauffman uses a number of different versions (cf. 1993: 37). In one version, individual fitness is plotted against individual genotype, i.e. the fitness or replication rate of particular genotypes. In another instance, the landscape is a graph of population mean fitness against the state of the population as measured by allele frequency or trait means (Barton 2005). The distance between genotypes or phenotypes, i.e. the extent to which they are similar or not, and their interactions define the rate of fitness in the landscape.

Kauffman (1993: 40–43) introduces a simple formal model of (rugged) fitness landscapes, i.e. the *NK*-model. There are a number of parts of a system *N* (e.g. genes in a genotype) that each makes a fitness contribution, which depends on the interaction with *K* other parts among the *N*. “The two main parameters of the *NK* model are the number of genes *N* and the average number of other genes *K* which epistatically influence the fitness contribution of each gene.” (Kauffman 1993: 42) Owing to the respective fitness contributions, the fitness of each genotype is assigned and as such a fitness landscape over the genotype space is created that can be visualized as a hypercube. In this version, fitness landscape models allow researchers to investigate the relationship between diversity, interaction and fitness of genotypes or phenotypes in their environment.

Kauffman’s work seems to resonate with social scientists because he phrases his ideas in such a generic way that they seem applicable to any kind of system—be it physical or social or combinations thereof. For example Kauffman (1995) suggested, as did others (e.g. Frenken 2006), that the model could be used to analyze the development of technologies in societies. Moreover, the visualizations as a means to enhance the accessibility of his ideas were received well. The visualization most often used in the social sciences—though, interestingly, not directly traceable in any of the sources in biology—represents a three-dimensional ‘landscape’ with the population represented on the *x*-axis (*N*) and interaction between the genes in the population on the *y*-axis (*K*). Fitness is then represented on the *z*-axis in the landscape. Each *NK*-configuration in the landscape relates to a possible individual fitness value. These are either assigned randomly, manually, or the value might be a function of the values taken from each dimension (Calcott 2008). Ultimately, this version of the *NK* model is considered applicable to many types of questions just because it “allows for a very general description of *any* system consisting of *N* components with *K* interactions between the components and in which there can be any number of states for each *N*” (Weber 1998: 135, italics in original). Within the social science fitness landscapes probed narratives about how actors (of any kind) had to interact with others in order to climb the peaks towards better outcomes or suffer drawbacks first in order to improve later (or countless other derivatives) (Gerrits and Marks 2014, 2015). Naturally, such ideas require a transfer between two different domains. We will discuss the dynamics of such a theory in the following section.

## Transfer

Weisberg (2007) distinguishes two ways of theorizing: Modelling on the one hand, and Abstract Direct Representation or ADR on the other. ‘Modelers’ theorize by constructing abstract models. By analyzing these models they learn about real-world phenomena. As such, modeling is an indirect way of learning about real-world phenomena. That is, researchers treat “models as an autonomous object” even if they do so “with a particular real-world phenomenon in mind” (Weisberg 2007: 224). To learn about real-world phenomena “the model must be *similar* to a real-world phenomenon in certain appropriate respects” (Weisberg 2007: 218, italics in original). ADR is another way of theorizing: ADR uses for example equations, graphs, pictures to represent empirical phenomena without the mediation of a model (Weisberg 2007: 2010). Researchers use this theoretical apparatus in a direct way to analyze empirical puzzles and their theoretical representations. That is, researchers “engaged in ADR analyse and represent the properties of a real-world phenomenon, suitably abstracted.” (Weisberg 2007: 226).

In the following, Weisberg’s approach is taken as a way to distinguish between different styles of theorizing. But it is not followed in his restrictive use of the term ‘model’. In the social sciences, the term ‘model’ is applied in a broader sense than in Weisberg’s examples for developing the above described distinction. Here, ‘model’ is used to capture the theoretical apparatus employed in both ways of theorizing.

With the goal of transferring a fitness landscape model to the social sciences in order to understand an actual real-world phenomenon, there is a restriction that the model should be able to process data obtained from the real world. This follows the common understanding of an explanation in social science, as argued by Hempel and Oppenheim (1948), that an accurate representation of the real world is necessary. In addition, the model should allow for unambiguous measurement of said data in order to avoid confusion about what is measured and how it is measured. It is appreciated that measurement in the social sciences is ambiguous by definition but that should not refrain researchers from being precise in defining what they would like to measure.

**Fig. 1:**
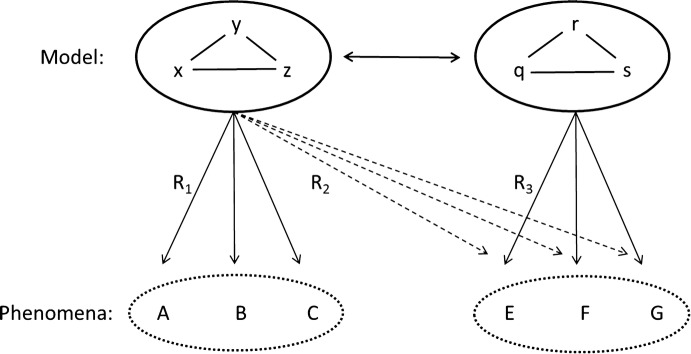
Knowledge transfer between source and target domain

It can be difficult to establish a representation that can describe the real world accurately (Knuuttila 2011). There is always a risk that in a strict transformation and application of the model the idealized model cannot accommodate certain features of the target domain, while in a rather free application any two things can be regarded as arbitrarily similar (cf. Bolinska 2013: 220). In order to transfer a model properly, it should achieve articulated awareness of the nature of the objects and relations of the social realm (cf. Woody 2004). As such, the transfer of a model is not a matter of simply copying its features as if the model is a blank template. Figure 1 illustrates this challenge.

The natural starting point for a transfer between domains is an existing model from the source domain, here the *xyz*-model. Mäki (2009: 33) argued that models can have epistemic and non-epistemic functions. The *xyz*-model has the epistemic function to represent phenomena in the source domain [A, B, and C]. In principle, there are two different ways how knowledge transfer between the source and target domain could happen: (1) an existing model explains the phenomena E, F, and G, i.e. claiming R_2_ is justified, and (2) the model is transformed to a new model (here from the *xyz*-model to the *qrs*-model) to explain the phenomena E, F, and G. In this second case a justification is needed why the *qrs*-model is an adequate representation (R_3_) for the target domain:

1. Scientists work with existing models to explain the target domain phenomena. Kuhn referred to this way of research as puzzle-solving, i.e. adding new phenomena to the set of the intended applications of a model (Kuhn 1970). Here, neither the syntactic patterns between the model’s variables nor its semantic interpretation is challenged. Rather, knowledge transfer proceeds by providing reasons to believe that the phenomena in the target domain are adequately represented (R2) by the *xyz*-model because of a high similarity between the targets.^1^
2. In the model transfer mode scientists explain the target domain phenomena with the transformed model. Do note that the syntactic structure of the model remains the same, but the semantic interpretation of the model is changed.^2^ That is, the original interpretation of the variables corresponds no longer to phenomena in the target domain and is modified accordingly. Hence, a new justification is needed why the model represents the target domain adequately. Once this is done, the model can be used by researchers for analyzing the phenomena in the target domain in the ADR-mode. This type of model transfer opens up further possibilities: The *xyz*-model and the *qrs*-model are syntactically isomorphic, as the target domain is addressed by finding functional equivalents to the variables of the original model suitable for the target domain. This enables researchers to learn indirectly about E, F, and G by manipulating and analyzing the *xyz*-model. Such learning through isomorphism is a typical feature of doing research in the modeler-mode.

We will now demonstrate this process by transferring and modifying the NK-model for the analysis of collective decision making. For the present purpose, the focus will be on the process of model transfer and the factors that influenced that transfer. This is done in two steps. First the basic properties of the target model will be discussed, and where and how they are modified. Then the main factors that lead to the modifications will be identified.

## Transfer of the original model

Collective decision-making concerns two or more actors coordinating over a certain issue in search of an improvement in their own situation, which sets the goal they want to reach. In the process, they will want to push through their own preferences and beliefs about that issue. Prime concern is *how* collective decisions are reached. Collective decision-making processes can be considered evolutionary processes (Axelrod 1984; 1986) and multiple authors have suggested that fitness landscapes can be of use to analyze such processes (e.g. Axelrod and Bennett 1993; Gerrits 2012; Rhodes and Dowling 2018; Room 2011, 2016; Teisman and Klijn 2008). In a nutshell, the argument goes that the model has explanatory value for collective decision-making processes because it allows the mapping of actors, their interactions and the outcomes in terms of fitness (Gerrits and Marks 2017). This is not to say that there is a unified use of fitness landscape models for the analysis of collective decisions. Quite the opposite: there is a wide variety of such models as each author reads, interprets and uses the model in his or her own way; ranging from a more metaphorical use of ‘turbulent’ landscapes to be climbed (Klijn and Koppenjan 2013) to the use of it as a tool to structure a game-theoretical situation of warring nations (Axelrod and Bennett 1993).

### Modifying the basic properties

The basic NK model that will be modified is the model where fitness is plotted against the number of parts *N* and the interactions among the parts *K*. The source version is clear in one dimension featuring a substantive gene with certain properties, which is adapted to the target domain to mean ‘content’. The other dimension in the source model represents the interaction between genes, which could be used in a straightforward fashion but has been defined under the header of ‘processes', by sticking to the social science nomenclature. This distinction corresponds with a classic one in collective decision-making (Cohen et al. 1972), with structure emerging from the combination of process and content.

#### N = Content = PSD

Substituting the N-dimension representing genes and its properties with a functional equivalent from the field of collective decision-making will be the first step in the model transfer. Instead of talking about genes we refer to actors and the content of their beliefs. This captures the epistemic side of the decision-making process. The content dimension features two idea categories that actors in the decision-making process hold about the issue. Firstly, actors perceive and frame the issue in their own actor-specific ways as there is rarely full consensus about the exact nature of a given issue (Fischer 1998; Rein and Schön 1996). This means each actor develops its own problem definition. Secondly, actors have certain specific ideas about the ways in which the issue should be solved, i.e. they formulate their own specific solution definitions. This is combined in *problem-and-solution definition* or *PSD*. The full problem and solution space in a complex issue is very likely to be highly-dimensional and any method would be overwhelmed by the qualitative differences between each PSD. The PSDs are therefore scaled to the number of problem and solution definitions for each actor. The narrowest PSDs consist of only one problem definition without a solution definition, or vice versa. The broadest PSDs are those that contain many different problem and solution definitions. The number of definitions in the different actors’ PSD ranges from one definition to the maximum number of definitions observed in a given field. These numbers are scaled to fractions, ranging from 0 to 1, where 1 represents the maximum number of elements in a definition coded for a certain actor in that specific field, and all others PSDs are fractions of that maximum. This makes the scale dynamic and fitting to the definitions present in a particular field. In other words:

$${\text{every }}\;{\text{actor}}\;i\;{\text{has}}\;PSD_{i} = \frac{{actual\; \# \;elements\; PSD_{i} }}{maximum \;\# \;elements \;PSD},\quad {\text{where}} \;PSD_{i} \in \left[ {0, \, 1} \right].$$

#### K = Process = c_score

The *K*-dimension concerns the interaction between genes in the source model, and is measured in terms of frequency (or degree of interrelatedness, conf. Levinthal and Warglien 1999: 350). To find a substitute for this variable in the realm of collective decision-making we have to define a variable that captures the interaction structure between the agents enabling them to share information. This causes a problem in the social realm, as interaction frequency is hardly informative because it cannot differentiate between qualitatively different interactions. On top of that, the real world sees a strong relationship between content and interaction, with the latter being a consequence of the (perceived) distance, coalition forming, collaboration, beliefs, friendships, et cetera (Marks 2002; Marks and Gerrits 2018). Actors will decide with whom to cooperate or align, or, conversely, whom to avoid or even to contravene, based on their perceptions, strategies, perceived distances and motives of those other actors (e.g. March 1994). As such, there is no independence between content and interaction. Instead of frequency, the degree of connectedness between actors has been opted for. *Connectedness* is the number of actual links in a network as rate of the number of possible links, i.e. the connectivity measure in evolutionary network biology (Proulx et al. 2005), which is a measure known as density in social network analysis (Tichy et al. 1979). This is denoted *c_score*. A complicating factor is that social reality doesn’t start from a green-field situation (as is the case in fitness landscape simulations where starting conditions are simply randomized). Therefore the history of the actors involved, such as sense of belonging, history of cooperation, et cetera, has to be factored in Marks (2002), Peyton-Young (1998), Sugden (2005). As an initial condition, every actor *i* is connected to all other actors to a certain degree; that is:

$$c\_score_{i} \left( {t_{0} } \right) = \frac{{actual\; connections_{i} }}{maximum \;\# \;connections},\quad {\text{where}}\;c\_score_{i} \in \left[ {0, \, 1} \right]$$

During the collective decision-making process, actors will find out which elements of their PSDs are (dis)similar to elements of other actor PSDs. Actors’ connectedness with others is partly informed by the extent to which certain problem and/or solution definitions converge or diverge. Conversely, convergence or divergence in PSDs is partly informed by the perception of the position of, and interaction with other actors, i.e. actor connectedness informs the PSD’s of actors. This mutual informing is the structural connection between two dimensions. It means that the link between actors and their respective PSDs will become weaker or stronger due to the (dis)similarity in content of elements in PSDs. Hence, a qualitative adjustment based on content (similar elements of the PSDs) enters, attributing weight (w) to the *c_score* for every actor. The change brought about by the similarity of elements in PSDs means that every actor *i* is now connected as follows:

$$c\_score_{i} \left( t \right) = w_{i} \times \frac{{actual \;connections_{i} }}{maximum \;\# \; connections};$$

$${\text{where}}\;w_{i} = \frac{{\# \;actual\; similar \;elements \;PSD_{i} }}{maximum\; \# \;similar\; elements \;PSD},\;{\text{and}}\;w_{i} \wedge c\_score_{i} \in \left[ {0, \, 1} \right]$$

The position of actors in the field may change due to the weight. Certain actors will be more isolated, while others will have a central position, but also certain actors can become more closely linked to each other than they are to rest of the network forming a cluster within the network. This basic set-up allows mapping the actors involved in a certain issue relative to each other. When engaging in collective decision-making processes, they shift in both dimensions as they opt to cooperate, or not, with certain actors and as they try to align their PSD’s, or avoid such alignment

#### Fitness

In the social realm one can obtain fitness at some point and lose it elsewhere without losing overall fitness, so a simplistic dichotomy between survival and extinction doesn’t work well there (for a similar discussion in biology see Gavrilets 2010; Reiss 2007). In addition, the social realm sees people doing things without any directly observable gains, e.g. helping out at a homeless shelter. It is therefore better to speak of inclusive fitness, which denotes the effect of an actors’ actions on its own fitness and that of others directly related to this actor (Grafen 2006; Hamilton 1964; West et al. 2011), or, conversely, that others can have an impact on the fitness of that particular actor, i.e. neighbor-modulated fitness. In collective decision-making it boils down to who gets what, when and how (Lasswell 1936), i.e. who manages to realize the (highest number of) elements in its PSD. That is, collective decision-making concerns actors trying to achieve a better *fit* with the demands and pressures from the environment. This is not a static environment and an actor’s ability to get closer to its goal depends on its position relative to other actors, and not just on its own intentions or deliberate planning but also on what others connected to that actor do. In other words, fitness is defined as the *probability* of an actor achieving its desired goals as defined in its PSD relative to all other actors; i.e. *relative fitness*.

Inevitably, fitness values need to be attributed in hindsight when it has become clear how and which actors managed to get (parts of) their PSD realized within the specific situation. As such, fitness is treated as an independent dimension in the model because there is no a priori clear causal idea about what combinations of content and process bring forth fitness gains or losses. That is, the fitness attribution is done by the researchers based on the data; i.e. the success probability to each actor’s configuration of PSD and the weighed *c_score* in relationship to that of others as derived from the interpretation of the data. In modeling terms, the fitness value (*f*) is attributed to the specific configuration of *PSD* and the weighted *c_score* for that actor, where *v* is the value attributed by the researchers:

$$f_{i} = v\left( {PSD_{i} , c\_score_{i} } \right)\quad {\text{for }}\;{\text{each }}\;{\text{actor}}\;{\text{ i,}}\;{\text{ where }}\;f_{i} \in \left[ {0, \, 1} \right].$$

Note that this assigning of fitness doesn’t require a priori assumptions about the composition of the (groups of) actors involved, the power distribution among them, the interests they represent et cetera. The question who is more likely to obtain fitness is an empirical one.

### Model transfer

**Fig. 2:**
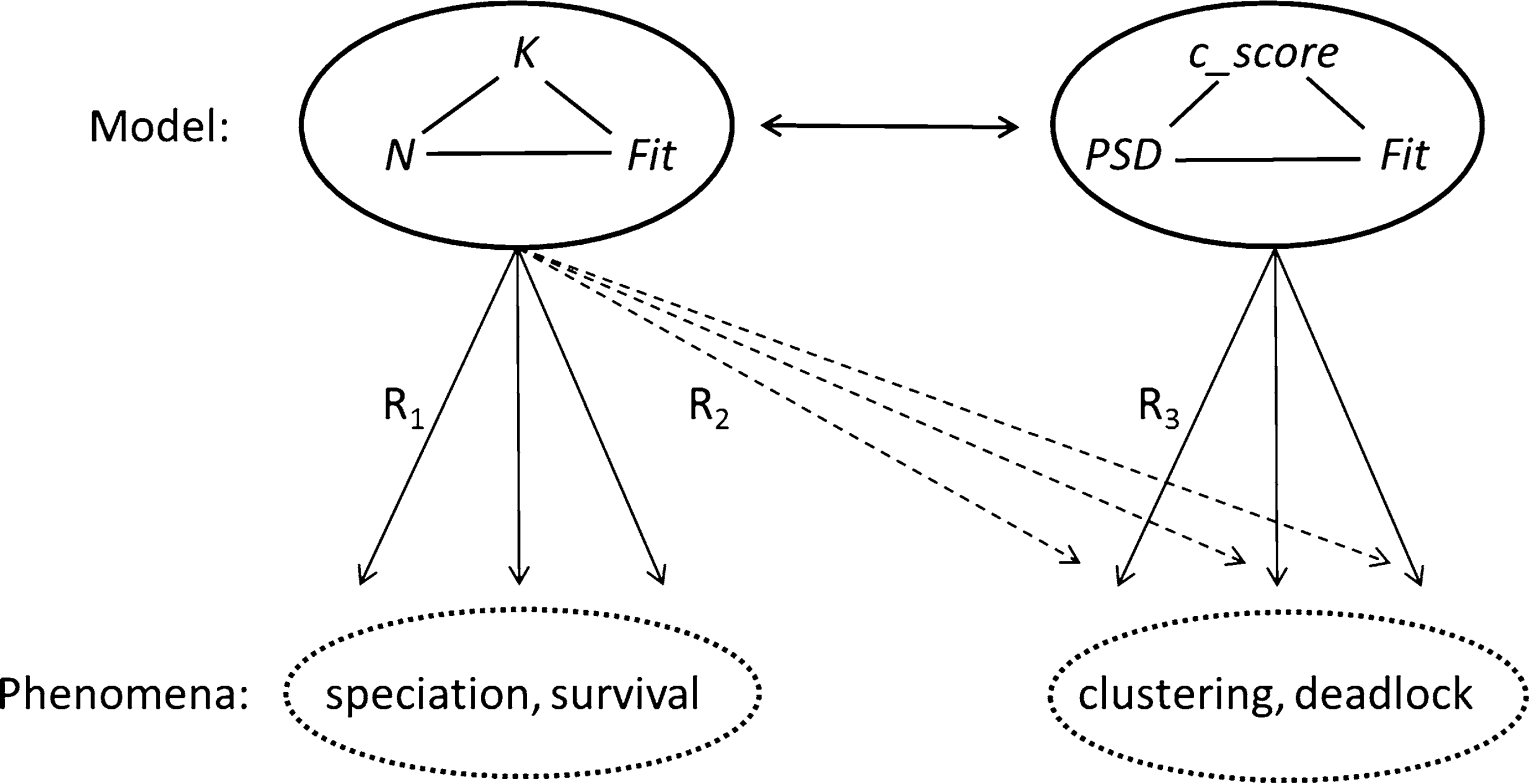
Actual knowledge transfer between biology and social sciences (Note: the phenomena mentioned in the figure are just examples in the respective domains)

The model above shows that there was a need to alter the semantics of the source model substantially. Albeit just a basic model, it already had to be changed in all three dimensions in order to suit the target domain. These modifications didn’t affect the syntactic structure of the model, however. Figure 2 shows the transfer as described above in terms source and target. As shown in the previous section, the *xyz*-model had to be transformed into the *qrs*-model to gain insights into collective decision-making: i.e. *N*, *K* and *Fit* into *PSD*, *c_score* and *relative fitness* (visualized in Fig. 2).

The R_2_ relation in Fig. 2 is not justifiable. However, the R_3_ relation is justifiable, owing to the following arguments. Human agency creates a considerable difference between biology and the social sciences. The reflexive capacities of humans—the ability to anticipate, forecast, reflect, reason, and learn—mean that they have a considerable range of behavioral responses to choose from. Moreover, social scientists may be able to obtain direct access to the individual motives for certain responses. Here biology and the social sciences diverge. This requires changes to the semantics of the model because:

1. It must account for the large range of possible actions, and
2. It must connect actions to a probabilistic take on fitness attainment: i.e. fitness is an independent component.

Collective decision-making processes have different properties than e.g. the study of genotype–phenotype combinations. This has consequences for the operationalization of the various dimensions, examples of which were shown in the model above. Therefore, the model features:

(3) A fitness range that does justice to a social equivalent of fitness, i.e. relative fitness.

All dimensions are revised in order to do justice to the complexities of the social realm. Having to change all the dimensions as well as the operationalization of those dimensions leads to issues of measurement. Many (if not all) fitness landscape models in biology rely on mathematics as well as simulations to do the work. This is much less so in the case of the social sciences where empirical (fuzzy) data play a pivotal role. There are considerable debates about the proper epistemology when it comes to assessing social empirics, as most social scientists will agree. Indeed, some will even contest the terms ‘measurement’ or ‘assessment’ and would rather opt for alternatives from constructivist schools. Following the common social sciences understanding of explanations (see Hempel and Oppenheim 1948) they require:

(4) Changes to the model’s measures, as the social sciences standard model of explanation demands that the premises of an explanation have to be empirically significant in the context of social sciences and valid.

Assuming that R_3_ is justifiable given the modifications 1–4, the *PSD, c_score*-model can be used for analyzing the target domain in the ADR-mode. Moreover, as the transfer left the syntactic structure of the model intact, additional ways of gaining insights into ‘clustering', ‘deadlocks', et cetera [i.e. E, F and G] open up.

## Conclusion

The paper set out to demonstrate that the transfer of the biological fitness landscape model to the social sciences is not a straightforward matter. The specific characteristics of the target domain meant that the semantics of the original model could not be retained, even though the syntactic structure could remain in place. Generally speaking, using models from outside impermeable disciplinary boundaries raises the problem for researchers to find functional equivalents to variables that work in the source domain. It may very well be the case that such equivalents are not found. However, the attempt at a transfer forces researcher to think about correspondences and dissimilarities between different disciplines. This constitutes an epistemic value by and of itself.

It is demonstrated that the transfer from biology to the analysis of collective decision-making is influenced by four major considerations. There is a trade-off in the transfer of the fitness landscape model from biology into the social sciences: the more the model is tailored towards the target domain, the more exploratory and explanatory power it will have, the less it will resemble the original model. If anything, it will put to rest the claims by Kauffmann (and some others) that fitness landscapes have universal explanatory value or constitute meta-frameworks for life in all its diversity. However, the model can be used in other domains when transferring the syntactic structure of the model only, leaving out the semantics. The model can then be used (1) to identify patterns in the interactions between actor (groups) who share a (dis)similar worldview, (2) to compare those patterns in respect to their relative fitness and (3) to ascertain those parts of the interaction structure where social scientists could carve out those mechanisms driving the emergence of that structure with a framework that is more geared towards identifying the mechanisms at play at the micro-level.

Since much of the theoretical knowledge about the original model depends on its syntactic structure only, this knowledge is easily transferable to the new model. But as the two models are isomorphic, it is possible to learn about the target domain by manipulating and analyzing the original *NK*-model in the modeler-mode. This opens the window for benefiting from the existing strand of theoretical literature about fitness landscape models and the various possible patterns and relations therein from the source domain to learn about the target domain in both direct and indirect ways.

## Footnotes

1 To avoid some possible misunderstandings: by using the terms ‘syntactic’ and ‘semantic’ we do not refer to the syntactic or semantic view on models as discussed in philosophy of science (Frigg and Hartmann 2017). Rather, these terms are used to differentiate between syntactic elements of a model, referring to the structural relations between the variables, and the intended interpretation of the model’s variables.

2 Even smaller changes in the syntactic structure may be justified, but there is certainly a threshold up to which the similarities between the models become too small to call it still a model transfer.
